# Interleukin-6, a reliable prognostic factor for ischemic stroke

**Published:** 2014-04-03

**Authors:** Sheyda Shaafi, Ehsan Sharifipour, Rouhollah Rahmanifar, SeyedShamseddin Hejazi, Sasan Andalib, Masoud Nikanfar, Behzad Baradarn, Robab Mehdizadeh

**Affiliations:** 1 Department of Neurology, School of Medicine, Tabriz University of Medical Sciences, Tabriz, Iran; 2 Department of Neurology, School of Medicine, Qom University of Medical Sciences, Qom, Iran; 3 Neurosciences Research Center, Tabriz University of Medical Sciences, Tabriz, Iran; 4 Department of Immunology, School of Medicine, Tabriz University of Medical Sciences, Tabriz, Iran

**Keywords:** Interleukin-6, Inflammatory Factors, Ischemic Stroke, Stroke Severity, Stroke Outcome

## Abstract

**Background:**Interleukin-6 (IL-6) is one of the inflammatory mediators characterized by elevated levels in ischemic stroke (IS) patients. The present study set out to assess the role of IL-6, as a marker for inflammation, in the severity and prognosis of acute IS.

**Methods:**In a cross-sectional descriptive study, 45 patients with acute IS were selected. Patients with their first day of stroke were included in the study. National Institutes of Health Stroke Scale (NIHSS) and the modified Rankin Scale (mRS) for stroke severity were evaluated on Days 1, 5, 90, and 365. Serum IL-6 level was measured by enzyme-linked immunosorbent assay (ELISA) on days 1 and 5.

**Results:**In the present study, 45 patients with a mean age of 77.6 ± 4.9 including 32 (71%) men and 13 (28.9%) women were studied. Death occurred in 2 (4.4%) patients before discharge from the hospital; the others, be that as it may, followed the study until Day 365 with a mortality rate of 6 (13.3%). A positive significant correlation was found between IL-6, and NIHSS and mRS of the patients from the time of admission to the end of the follow-up period (P < 0.001, r = 0.6). Moreover, there was a significant correlation between IL-6 and infarction size in brain magnetic resonance imaging (MRI) scan (P < 0.001, r = 0.7).

**Conclusion:**The evidence from the present study suggests that IL-6 contributes to determination of severity of ischemic stroke. In addition, IL-6 concentrations affect clinical outcomes in ischemic stroke.

## Introduction

Stroke is one of the main public health concerns and the main cause of long term disability.^1^^,^^2^ It has been known as the second most common cause of mortality throughout the world.^3^^,^^4^ Scientific evidences suggested inflammation as a key factor in the pathological response of stroke. ^5^^-^^7^  The majority of inflammatory reactions to acute cerebral ischemia are mediated by cytokines which increase in the central nervous system (CNS) and the systemic circulation in patients with acute ischemic stroke (IS).^5^^,^^8^^-^^10^Interleukin-6 (IL-6) is a crucial inflammatory factor in that its significant increase was observed in stroke patients shortly following the ischemic event and serves a vital role as a messenger molecule between leucocytes, the vascular endothelium, and parenchyma resident cells. IL-6 is likely to bring about an array of diverse and competing effects encompassing anti-apoptotic, pro-proliferative, growth-inhibitory, and differentiation-inducing effects depending on the cellular context. There was little agreement on the source of the early surge in circulating IL-6 levels in stroke for some time. ^11^^-^^14^ 

The prediction of death or disability (poor outcome) subsequent to ischemic strokehas been an area of interest for neuroscientists. It was shown that statistical models, predicated based on clinical variables, namely age or neurological impairment, created similar predictions of poor outcome to that of experienced stroke physicians. Additionally, according to bedside clinical examination, it was demonstrated that biomarkers of the processes that are active in ischemic stroke might add predictive power to these simple statistical models.^15^^,^^16^ Although numerous preceding published studies suggested an association between inflammatory mediators, such as IL-6, and brain damage, and stroke progression and severity, the associations found in group data, unless very strong, do not constantly fulfill better predictions of outcome in IS patients.^9^^,^^13^^,^ ^15^^,^^17^^,^^18^ The present study was designed to investigate the relationship between serum level of IL-6, and the stroke outcome and disability as assessed by National Institutes of Health Stroke Scale (NIHSS) and modified Rankin Scale (mRS) scoring at the time of admission, Day 5, month 3, and Year 1 following the acute ischemic stroke.

## Results

***Demographic findings***

The mean age of stroke patients was 77.68 ± 4.91 years; ranging from 65 to 85 years. In addition, 35 (77.7%) patients had stroke in MCA territory, 8 (17.7%) in ICA territory, and 2 (4.4%) in ACA territory. The mean IL-6 plasma concentration was found to be 42.92 ± 72.2 pg/ml (ranging from 0 to 367.80) and 56.91 ± 82.63 pg/ml (ranging from 0 to 444.6) on Day 1 and Day 5, respectively. The mean NIHSS on hospitalization day and on Day 5 was 10.8 ± 5.65 (ranging from 2 to 20) and 10.1 ± 5.60 (ranging from 2 to 22), respectively. The mean NIHSS on the 3^rd^ month and 1^st^ year was 7.02 ± 5.32 (ranging from 0 to 18) and 3.86 ± 3.02 (ranging from 0 to 12), respectively. There were 41 and 37 participants on the 3^rd^ month and 1^st^ year, respectively. MRI showed a mean infarct of 19.26 ± 10.07 cm^3^ (ranging from 6.5 to 45). The mean mRS on Day 5, Day 90, and Year1 was 3.93 ± 1.19 (ranging from 0 to 18), 3.17 ± 1.65 (ranging from 0 to 6), and 2.31 ± 2.10 (ranging from 0 to 6), respectively. The mortality rate was found to be 17.7%, which occurred in 2 (4.4%) patients before discharge from the hospital, 2 (4.4%) patients from time of discharge until Day 90, and 4 (8.9%) other patients from months 3 to 12.

***Association of IL-6 level, NIHSS, mRS, and other variables***

As can be noted in table 1, NIHSS on Days 1, 5, and 90, and year 1 was significantly associated with the level of IL-6 (all with P ≤ 0.001). There was also a significant association between mRS on Days 5, and 90, and Year 1 and the level of IL-6 (all with P ≤ 0.001). The infarct size was shown to be significantly associated with the level of IL-6 (all with P ≤ 0.001). However, there is no difference between the serum IL-6 level of patients with stroke in different vascular territories (ICA, MCA, and ACA).

**Figure 1:**
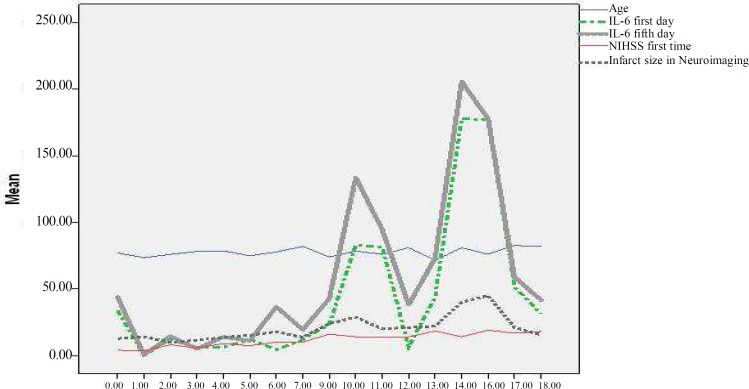
Comparison of association of various variables with NIHSS on month 3

**Figure 2:**
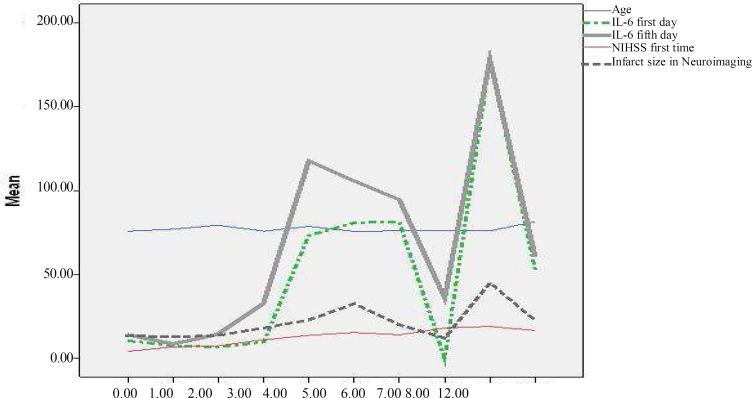
Comparison of association of various variables with NIHSS on Year 1

**Figure 3:**
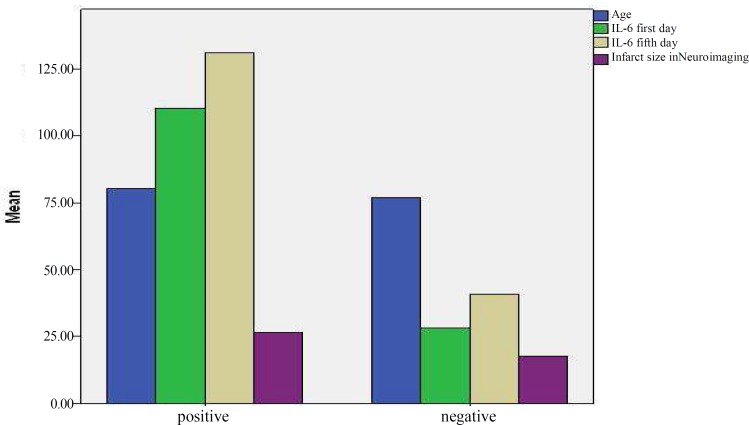
Comparison of association of various variables with presence and lack of mortality

Table 2 illustrates that NIHSS on Day 1 was significantly associated with NIHSS on Days 5, and 90, and Year 1 (all with P ≤ 0.001). There was also a significant association between NIHSS on Day 5 and NIHSS on Day 90 and at Year 1 (all with P ≤ 0.001). NIHSS on Day 90 was found to be significantly associated with NIHSS at Year 1 (P ≤ 0.001). Moreover, mRS on Day 5 was significantly associated with NIHSS on Days 0, 5, and 90, and at Year 1 (all with P ≤ 0.001). In addition, mRS on Day 90 was shown to be significantly associated with NIHSS on Days 0, 5, and 90, and at Year 1 (all with P ≤ 0.001). There was a significant association between mRS at Year 1 and NIHSS on Days 1, 5, and 90, and at Year 1 (all with P ≤ 0.001). Age was significantly associated with NIHSS on Day 90 and the infarct size was found to be associated with NIHSS on Days 1, 5, and 90, and at Year 1. Furthermore, blood levels of IL-6 were significantly higher in the stroke patients who died. Comparison of association of various variables with NIHSS on Month 3 and on Year 1 is shown in figure 1 and 2, respectively. Table 3 shows the 25, 50, and 75 percentiles of IL-6 levels in the patients with and without mortality occurrence and figure 3 demonstrates the comparison of association of various variables with presence or lack of mortality. 

**Table 1:** Association of IL-6 level with NIHSS, mRS and other infarcts

	**IL-6 level on day 1**		**IL-6 level on Day 5**	
	**Spearman’s rho**	**P**	**Spearman’s rho**	**P**
NIHSS on Day 1	0.719	≤ 0.001	0.876	≤ 0.001
NIHSS on Day 5	0.718		0.864	
NIHSS on Day 90	0.593		0.745	
NIHSS at Year 1	0.568		0.741	
mRS on Day 5	0.660		0.806	
mRS on Day 90	0.710		0.782	
mRS at Year 1	0.601		0.672	
Infarct size in MRI scan	0.737		0.740	

Spearman’s rho; Spearman's rank correlation coefficient

**Table 2:** Association of NIHSS with mRS and other infarcts

	**NIHSS on day 1**		**NIHSS on day 5**		**NIHSS on day 90**		**NIHSS at Year 1**	
	**Spearman’s** ** rho**	**P**	**Spearman’s ** **rho**	**P**	**Spearman’s** ** rho**	**P**	**Spearman’s** ** rho**	**P**
NIHSS on Day 5	0.983	≤ 0.001	1	-	0.932	≤ 0.001	0.871	≤ 0.001
NIHSS on Day 90	0.888	≤ 0.001	0.932	≤ 0.001	1	-	0.922	≤ 0.001
NIHSS at Year1	0.849	≤ 0.001	0.871	≤ 0.001	0.922	≤ 0.001	1	-
mRS on Day 5	0.928	≤ 0.001	0.917	≤ 0.001	0.846	≤ 0.001	0.827	≤ 0.001
mRS on Day 90	0.882	≤ 0.001	0.911	≤ 0.001	0.980	≤ 0.001	0.895	≤ 0.001
mRS at Year1	0.829	≤ 0.001	0.824	≤ 0.001	0.863	≤ 0.001	0.953	≤ 0.001
Age	0.060	0.695	0.186	0.0232	0.302	0.055	0.166	0.328
Infarct size	0.620	≤ 0.001	0.620	≤ 0.001	0.0553	≤ 0.001	0.459	0.004

Spearman’s rho; Spearman's rank correlation coefficient

**Table 3:** The 25, 50, and 75 percentiles of IL-6 levels in patients with and without mortality occurrence

	**Death**	**Percentile of 25**	**Percentile of 50 (median)**	**Percentile of 75**	**P**
IL-6 level on Day 1	Positive	14.50	85.15	165.67	0.012
	Negative	0.55	8.90	22.33	
IL-6 level on Day 5	Positive	26.81	89.50	192.62	0.010
	Negative	1.05	19.94	63.25	

**Table 4:** Prediction of NIHSS based on level of IL-6

	**NIHSS on Day 1**		**NIHSS on Day 5**		**NIHSS at month 3**		**NIHSS at Year 1**	
	**β**	**P**	**β**	**P**	**β**	**P**	**β**	**P**
IL-6 level on Day 1	0.047	≤ 0.001	0.069	≤ 0.001	0.064	≤ 0.001	0.031	0.002
IL-6 level on Day 5	0.045		0.070		0.063		0.030	≤ 0.001

**Table 5:** Prediction of mRS based on level of IL-6

	**mRS on Day 5**		**mRS on Day 90**		**mRS at Year1**	
	**β**	**P**	**β**	**P**	**β**	**P**
IL6 level on Day 1	0.009	≤ 0.001	0.014	≤ 0.001	0.015	≤ 0.001
IL6 level on Day 5	0.009		0.013		0.013	

Tables 4 and 5 show results for prediction of NIHSS and mRS based on level of IL-6 using linear regression analysis.

Moreover, the logistic regression analysis for the prediction of mortality based on IL-6 level resulted in the following formulas:


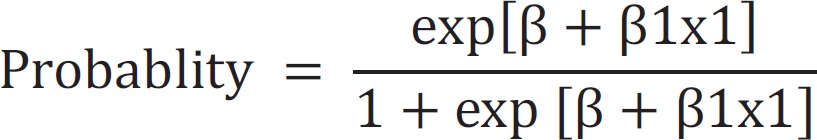



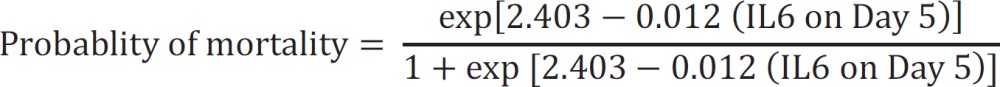



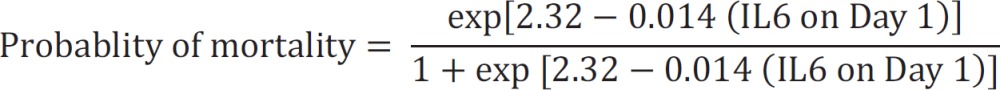


## Discussion

In recent years, there has been a propensity to understand the role of inflammatory factors, especially IL-6 in the stroke. The present study assessed the association of IL-6 with the severity and prognosis of patients with acute ischemic stroke and showed that increased level of this inflammatory marker in the acute stroke phase is associated with the severity of neurological damage in either clinical or imaging aspects. It was also shown that increased level of IL-6 on Days 1 and 6 is associated with mortality rate, functional disability, and performance status (in month 3 and Year 1). This association was in accordance with other influencing factors in this regard, such as age, neurological impairment after acute events, or infarction volume in neuroimaging. 

A considerable amount of literature has been published on the role of inflammatory markers in stroke. By way of illustration, molecular markers of inflammation were demonstrated to be useful for the management of ischemic stroke patients during the acute phase and for prognosis and prevention of risk. To clarify, inflammatory cytokines, such as IL-6, tumor necrosis factor alpha (TNFα), and adhesion cell molecules, contribute to early neurological deterioration and infarct volume.^   19 ^ Evaluation of stroke patients following acute ischemic stroke on admission and on the 28^th^ day subsequent to the onsetalsoshowed that IL-6 may predict not only the severity of lesions but also the outcome of patients.^   20 ^Elsewhere, assessment of 1-month outcome of stroke, by means of the Barthel index, demonstrated initial cerebrospinal fluid interleukin-6 (CSF IL-6) measured 6 hours after onset of stroke and nitrate levels in cerebrospinal fluid were significant for functional outcome of stroke at 1 month.^   21 ^Combination of circulating IL-6 and heart-type fatty acid binding protein level was also shown to have an additive clinical value for the prediction of ischemic stroke outcome.^   22 ^

In another study, by assessment of initial ischemic lesion size and neurological dynamics during 1 month of acute brain ischemia, high plasma level of IL-6 in the acute phase of stroke was shown to be a strong predictor of poor outcome for aged rather than for younger patients.^   23 ^ Clark et al. measured plasma levels of IL-6, fibrinogen, white blood cells (WBCs), and serum albumin as acute phase response (APR) in 4±2 days of onset in ischemic stroke patients. The authors defined standard clinical predictors as initial NIHSS, infarct size on CT, and the Glasgow scale. It was concluded that the initial APR was highly correlated with 6-month stroke recovery and this approache was in correlation with standard clinical predictors.^   24 ^ In another study, inflammatory markers such as monocyte chemotactic protein-1 (MCP-1), matrix metalloproteinase-9 (MMP-9), and tissue inhibitor of matrix metalloproteinase-1 (TIMP-1), interleukin-6 (IL-6), C-reactive protein (CRP), and the brain damage marker S100B were demonstrated to show significantly different time courses depending on stroke outcome. Despite the fact that the levels of IL-6, MCP-1, and MMP-9 increased a few hours subsequent to symptom onset, CRP and S100B gradually increased starting at 12-24 hours. IL-6, MCP-1, TIMP-1, and S100B were also shown to be independently associated with clinical 90 days outcome scores (mRS and NIHSS) at certain time points.^   25 ^

The present study produced results corroborating the findings of a great deal of the abovementioned research in that there was an association between the plasma level of IL-6 on Days 1 and 5 and the initial severity of disease, infarct volume found by neuroimaging, performance status, and the severity of damage at month 3 and Year 1. Furthermore, logistic regression analysis showed the formulas for the prediction of mortality based on IL-6 level. The effect of high-sensitivity IL-6 as a possible biomarker at the early stages of acute stroke in order to identify patients who were at a high risk for 12-month mortality was evaluated by Shenhar-Tsarfaty et al.^   26 ^ The authors confirmed the clinical potential of using high-sensitivity IL-6 as an early signal for acute ischemic stroke survival and demonstrated a clear cut point (6.47 pg/ml) for patients at a high risk.^   26 ^In the present study, the mortality rate was significantly higher in patients with higher serum level of IL-6.

On the contrary, however, there are some reports unable to demonstrate the role of IL-6 in stroke patients. A significant association between the severity of neurological deficit at admission, the diagnostic subtype, and the inflammatory variables was shown by Tuttolomondo et al.^   27 ^ In addition, ischemic stroke patients with cardioembolic subtype experienced significantly higher median plasma levels of TNFα, IL-6, IL-1β, notwithstanding significantly lower median plasma levels in the lacunar subtype.^   27 ^In the current study patients with stroke in the large vessels territory of anterior brain circulation were exclusively included and no significant difference was found in serum IL-6 between different territories of involvement. 

In a study carried out by Whiteley et al. following adjustment of stroke severity and age, only IL-6 and N-terminal pro-brain natriuretic peptide were significantly associated with poor outcome. However, neither IL-6 nor N-terminal pro-brain natriuretic peptide showed sufficient predictive power to be of clinical value.^   15 ^ In a 4-year prospective cohort study of inflammatory markers, higher levels of IL-6, CRP, and fibrinogen were shown to be associated with an increased risk of recurrent vascular events, vascular death after stroke, and nonvascular causes of death. However, it was concluded that inflammatory markers do not serve a causal role, particularly in the generation of recurrent vascular events subsequent to stroke.^28^Whiteley at al. confirmed that increased levels of acute inflammatory response markers after stroke (i.e. IL-6, CRP, fibrinogen, white cell count, and glucose) were associated with poor outcome, although the addition of such markers to a previously validated stroke prognostic model failed to improve the prediction of poor outcome.^   29 ^ Welsh et al. evaluated clinical status and 16 biomarkers in 24 hours of onset in acute patients with ischemic stroke and showed that CRP, IL-6, and fibrin D-dimer had the strongest univariate associations with poor outcome. However, D-dimer and CRP, exclusively, were independently associated with poor outcome in acute ischemic stroke in a multivariable mode.^30^ Oto et al. assessed levels of IL1beta, IL-6, IL10, TNF-alpha, catecholamines, epinephrine, and norepinephrine and found that in ischemic stroke plasma cytokines and catecholamines were not predictors of neurological outcome at 1 month. However, in the early phase of hemorrhagic stroke, high levels of IL-6 showed a poor neurological outcome.^   31 ^

The unique feature of this study was the attempt based on the IL-6 changes in the new patients of acute IS in the large vessel territory of anterior brain circulation, using 2 times IL-6 serum assessment on Days 1 and 5, to evaluate the correlation of these levels with different aspects of acute IS (such as early disease severity, infarction volume, functional status (on Days 5, 90, and 365), and mortality rate during 1 year follow-up) and compare the effect of IL-6 with other influencing factors in this regard (such as age, severity of stroke on admission, and infarct volume in neuroimaging). In conclusion, the results showed that IL-6 has a significant correlation with all these aspects of IS and this inflammatory marker is in agreement with other standard predictors of IS. 

Finally, it is hard to escape the obvious conclusion from the present study that plasma level of IL-6 is of value in determining the extent of ischemic stroke and associated with mid-term outcome and mortality rate of the stroke patients. However, a more broad research is also needed to determine the precise role of inflammatory factors in stroke. Moreover, a limitation of the present study was its relatively small sample size. Thus, it would be interesting if further investigation with a larger sample size is carried out.
